# Isolation of Mesophyll Protoplasts from Mediterranean Woody Plants for the Study of DNA Integrity under Abiotic Stress

**DOI:** 10.3389/fpls.2016.01168

**Published:** 2016-08-15

**Authors:** Elena Kuzminsky, Roberta Meschini, Serena Terzoli, Liliana Pavani, Cristian Silvestri, Zineb Choury, Giuseppe Scarascia-Mugnozza

**Affiliations:** ^1^Laboratory of Forest Biotechnology, Department for Innovation in Biological, Agro-food and Forest systems, University of TusciaViterbo, Italy; ^2^Laboratory of Molecular Cytogenetic and Mutagenesis, Department of Ecological and Biological Science, University of TusciaViterbo, Italy; ^3^Laboratory of Tissue Culture and Biotechnology of Woody Plants, Department of Agricultural and Forestry Sciences, University of TusciaViterbo, Italy

**Keywords:** holm oak, protoplast isolation, SCGE assay, DNA integrity, abiotic stress

## Abstract

Abiotic stresses have considerable negative impact on Mediterranean plant ecosystems and better comprehension of the genetic control of response and adaptation of trees to global changes is urgently needed. The single cell gel electrophoresis (SCGE) assay could be considered a good estimator of DNA damage in an individual eukaryotic cell. This method has been mainly employed in animal tissues, because the plant cell wall represents an obstacle for the extraction of nuclei; moreover, in Mediterranean woody species, especially in the sclerophyll plants, this procedure can be quite difficult because of the presence of sclerenchyma and hardened cells. On the other hand, these plants represent an interesting material to be studied because of the ability of these plants to tolerate abiotic stress. For instance, holm oak (*Quercus ilex* L.) has been selected as the model plant to identify critical levels of O_3_ for Southern European forests. Consequently, a quantitative method for the evaluation of cell injury of leaf tissues of this species is required. Optimal conditions for high-yield nuclei isolation were obtained by using protoplast technology and a detailed description of the method is provided and discussed. White poplar (*Populus alba* L.) was used as an internal control for protoplast isolation. Such a method has not been previously reported in newly fully developed leaves of holm oak. This method combined with SCGE assay represents a new tool for testing the DNA integrity of leaf tissues in higher plants under stress conditions.

## Introduction

There are a lot of hazards connected to global change at a global scale, and air pollution is considered one of the single largest environmental risks because it leads to a multitude of adverse effects on human health and ecosystems (European Environment Agency [EEA], 2015). In particular, the concentration of ground-level ozone, one of the most risky air pollutants due to its strong oxidizing power, increases from cold regions to warmest regions, such as the Mediterranean region (Safieddine et al., 2014). Plant responses to O_3_ vary tremendously by species, genotype, and leaf age (Karnosky et al., 2007), but relatively little is known about the genetic control of the response and adaptation of trees to tropospheric ozone exposure (Street et al., 2011). Exploring the genotoxicity of some pollutants such as ground-level ozone in tree species could be crucial for the protection and conservation of Mediterranean ecosystems (Scarascia-Mugnozza et al., 2000). In that respect, holm oak (*Quercus ilex* L.) has been selected as the model plant to identify critical levels of O_3_ for Southern European forests, because of its very wide diffusion in natural ecosystems and in urban areas, as well as its high tolerance to air pollution (Alonso et al., 2008).

Due to constraints in obtaining suitable nucleoids (Collins, 2004; Ventura et al., 2013), plant tissues are considered difficult for the employment of single cell gel electrophoresis assay (SCGE), a routine procedure for the detection of genotoxicity in animal cells. SCGE or Comet assay is a gel electrophoresis performed at the single cell level. In the assay, single cells or nuclei are embedded in agarose on a slide and lysed with detergent and high salt concentration. This last step digests the cellular and nuclear membranes and allows the release of coiled DNA loops generally called nucleoids and DNA fragments. Electrophoresis at high pH results in structures resembling comets, which can be observed by using appropriate fluorescent stains; DNA fragments migrate away from the “head” into the “tail” based on their size, and the intensity of the comet tail relative to the total intensity (head plus tail) reflects the amount of DNA breakage (Olive et al., 1990; Tice and Strauss, 1995; Collins, 2004). The comet assay is a method for evaluating genotoxiticy that identifies substances that cause DNA damage. In particular, under alkaline conditions (pH > 13), the comet assay can detect single and double stranded breaks and alkali labile sites. Comet assay is a rapid and sensitive procedure applicable, in principle, to any organism or tissue from which analyzable single cell/nuclei suspensions can be derived and recently increased use of this method has been suggested in plant toxicology (Santos et al., 2015) and functional genomics studies (Brunner et al., 2014). In this context protoplasts could be considered a new source for obtaining nuclei from leaf tissues. Since the first isolation of plant protoplasts was reported more than 50 years ago (Cocking, 1960), this technology has been widely applied in order to study membrane biology, cellular processes and activities, somaclonal variations, and DNA manipulations (Eeckhaut et al., 2013). Applying protoplast technologies to develop pathogen resistant material is a novel approach which has highlighted its potentiality and the difficulties related to tree species at the same time (Jones et al., 2015). The physiological status of the tissues employed is crucial for obtaining viable protoplasts. Usually, the axenic *in vitro* growth facilitates the release of viable protoplasts starting from plant organs (leaves, stems, buds, and roots), seedlings and cell suspension cultures in contrast to *in vivo* and glasshouse-grown plants, which are influenced by contaminations and seasonal variations; specifically, Jones et al. (2012) demonstrated that phenylpropanoids confer resistance to enzymatic digestion of plant tissues as they lead to an increase of lignin and other polyphenols that harden the cell wall (2012) and inhibit cellulase activity (Ximenes et al., 2010). The age of the leaves and the time of the year, as well as abiotic and biotic stresses can affect polyphenols concentration (Rivas-Ubach et al., 2014) and this could be the reason why the removal of the cell wall is sometimes difficult and unpredictable (Jones et al., 2015). Although, a large yield of nuclei can be obtained from protoplasts (Gichner et al., 2008), the protocols for their isolation are available for a few species, mostly herbaceous, and they include different steps such as cell wall digestion, filtration, centrifugation, and separation of nuclei by density gradient centrifugation. In plant tissues, the SCGE technique is generally performed with a limited number of nuclei in comparison to animal studies, due to the difficulty to achieve a suitable amount of nuclei (Lanier et al., 2015). This is particularly true for mature leaves, which are also very interesting for testing the effects of air pollution, as they are the primarily affected tissues.

The establishment of a method for SCGE assay in Mediterranean plants, such as holm oak and white poplar, by using mesophyll protoplasts is the aim of the present work. To quantify DNA damage in isolated protoplasts, exposure to X-rays was performed.

## Materials and Methods

### Plant Material

#### Holm Oak

During spring and summer 2016, young shoots from a topped plant of 15 years-old (first and second growth fluxes, started on April, 4 and June, 16, respectively) were collected before leaf tissues hardened inside the urban park of Viterbo (Italy). The shoots (the bottom part put in water) were immediately transferred to the lab. Very young leaves (fourth leaf from the apex) and recently fully developed leaves (12th leaf from the apex) were analyzed. The material was handled within 1 h from sampling. The leaves were sterilized with 96% alcohol (1 min) and rinsed with sterile water thrice before performing the isolation procedures. The removal of trichomes from abaxial side (12th leaves only) of the leaf was necessary in order to avoid interference with the Comet assay and a specific method developed in *Geranium* was employed (Yerger et al., 1992). Briefly, the leaves were kept in ice, and the undersides were abraded by a previously chilled razor blade. This step ensured the removal of most of the trichomes, while the remainder were quickly vitrified in liquid nitrogen and were finally removed by placing the leaf in a test tube containing shredded dry ice and agitated with a vortex for 1 min.

#### White Poplar

The fourth leaf from the apex (*in vitro* rooted plants) of the 6K3 white poplar genotype (Abbruzzese et al., 2009) was employed as an internal control in each trial of protoplast isolation to check the efficiency of the enzyme solution.

### Isolation of Protoplast From Leaf Tissues

**FIGURE 1:**
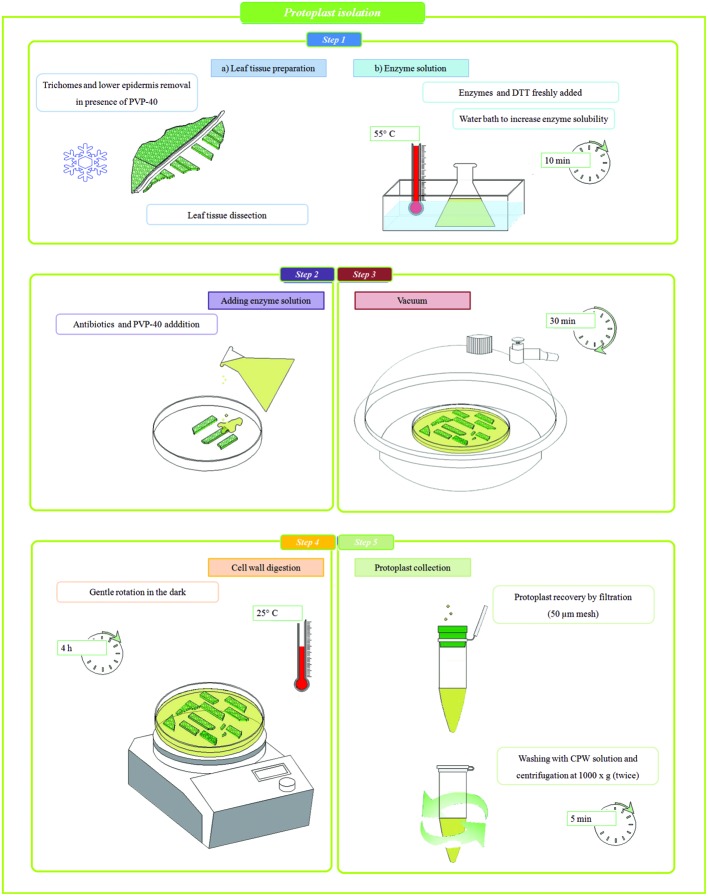
**Workflow of enzymatic method for mesophyll protoplast isolation**.

Mesophyll protoplasts of holm oak were obtained as follows. After trichome removal (12th leaf only), weighted leaves (~ 60 mg for the fourth leaf and ~ 60 mg for a portion from the middle part of the lamina for the 12th leaf) were scratched with a sterile scalpel into a cell and protoplast washing media (CPW) solution (**Table 1**), covering the lamina with a thin layer of Polyvinylpyrrolidone (PVP-40) powder. Immediately after, the leaves were cut (0.5 cm × 0.5 cm) excluding petiole and mid rib region and maintained for 5 min in CPW to remove debris. Then leaf tissue was dipped, inside a Petri dish (35 mm – Standard Line), in 2.5 ml of filtered (0.45 μm) enzyme solution (**Table 2**) prepared starting from Wakita et al. (1992) protocol set up for callus culture of *Quercus acutissima*. Briefly, mannitol (0.6 M), KH_2_PO_4_ (1 mM), NH_4_NO_3_ (5 mM), and sodium citrate (5 mM) were dissolved in distilled water and maintained at 4°C after the adjustment of pH value at 5.0. DTT (2 mM), cellulase Onozuka RS (2%) and macerozyme Onozuka R-10 (1%) were freshly added and the enzyme solution was put in a water bath at 55°C for 10 min in order to improve enzyme solubility (Yoo et al., 2007). Carbenicillin and cefotaxime (0.5% v/v), and PVP-40 (1% w/v) were freshly added to the Petri dishes before putting the samples in a vacuum environment for 30 min. The digestion was performed at 25°C for 4 h in the dark with gentle orbital shaking (50 rpm). Protoplast suspensions (1 ml) were filtered (CellTrics^®^ 50 μm mesh) into an Eppendorf tube and added with 1 ml of CPW before centrifuging at 1000 × g for 5 min (He et al., 2016). The protoplast pellet was re-washed by re-suspension and centrifugation in 2 ml of CPW. A workflow of the entire protocol is presented in **Figure 1**.

**Table 1:** Cell and protoplast washing (CPW) solution.

Components	Concentration
KH_2_PO_4_	0.2 mM
KNO_3_	1 mM
CaCl_2_.2H_2_O	10.1 mM
MgSO_4_.7H_2_O	1 mM
KI	0.96 μM
CuSO_4_.5H_2_O	0.16 μM
Mannitol	11%
BSA	0.1%

**Table 2:** Enzyme solution for isolating protoplasts.

Components	Concentration
KH_2_PO_4_	1.0 mM
NH_4_NO_3_	5.0 mM
Sodium citrate	5.0 mM
Mannitol	0.6 M
pH	5.0
**Freshly added**	
DTT	2.0 mM
Macerozyme Onozuka R-10	1.0%
Cellulase Onozuka RS	2.0%

Protoplast yield was evaluated by cell counting with a hemocytometer under an inverted light microscope and their viability was assessed using Trypan blue (TBE) assay (1:1 v/v). Then, the isolated protoplasts were analyzed by light microscopy and tested with the SCGE assay. Every time holm oak leaves were processed for protoplast isolation, a sample of *in vitro* white poplar genotype was used as an internal control, following the same procedure with the exception of scretching and PVP-40. All the steps were conducted aseptically.

### Irradiation Condition

Isolated protoplasts were X-irradiated with 3 Gy at 37°C with a 250 kV and 6 mA with a Gilardoni MGL 200/8 D X-ray apparatus at a dose-rate of 60 cGy/min in CPW medium.

### Single Cell Gel Electrophoresis Assay

The standard alkaline (pH > 13) SCGE, or comet assay, was performed as described earlier under visible fluorescent light (Tice et al., 2000). In short, 20 μl of the isolated protoplasts were mixed with 80 μl of 0.75% low melting-point agarose in PBS at 37°C and immediately pipetted onto a frosted glass microscope slide pre-coated with a layer of 1% normal melting-point agarose, similarly prepared in PBS. Two slides for each experimental point were then incubated in lysis solution (2.5 M NaCl, 10 mM Tris-HCl, 100 mM EDTA, pH 10, with 1% Triton and 10% DMSO freshly added) for 1 day at 4°C. After lysis, slides were placed on a horizontal electrophoresis unit containing fresh electrophoresis buffer (1 mM EDTA, 300 mM NaOH, pH 13) and incubated for 20 min to allow unwinding of DNA. Electrophoresis was then conducted for 30 min at 25 V and 300 mA at 4°C. Subsequently, the slides were gently washed three times in a neutralization solution (0.4 M Tris-HCl, pH 7.5) for 5 min. Slides were stained with 50 μl ethidium bromide (20 μg/ml) and covered with a cover slip. Stained nucleoids were examined at 400× magnification with an automatic image analyzer (Comet Assay III, Perceptive Instruments, UK) connected to a fluorescence microscope (Axioskop 2, Zeiss). To evaluate the amount of DNA damage, computer-generated tail moment (tm) values and percentages of DNA damage were used. For each experimental point, 100 cells were scored from two slides.

### Statistical Analysis

The analysis of the significance for comet parameters, of the effect of the X-rays versus the control samples was performed with the Student’s *t*-test (*ts*) for paired samples (*p* < 0.01).

## Results and Discussion

**FIGURE 2:**
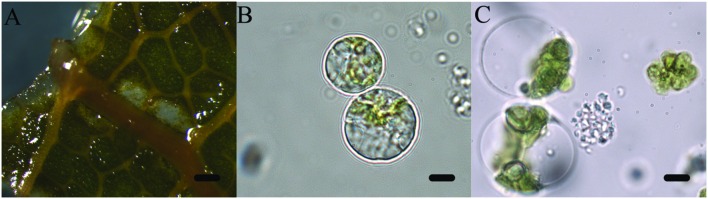
**Isolation of protoplasts from leaf tissue by enzymatic method. (A)** Digested holm oak leaf tissues after 4 h at 25°C (scale bar length: 6 mm); **(B)** Mesophyll protoplasts of holm oak from fourth leaf (scale bar length: 10 μm); **(C)** Mesophyll protoplasts of white poplar (scale bar length: 10 μm).

**FIGURE 3:**
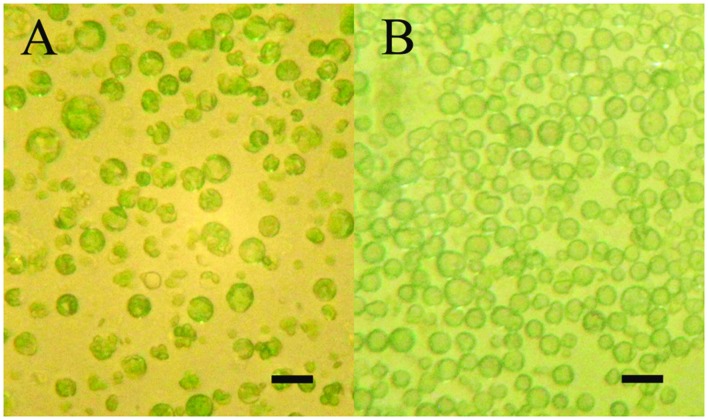
**Mesophyll protoplasts at inverted microscope at the end of digestion (4 h) into enzyme solution. (A)** White poplar (fourth leaf; scale bar length: 60 μm); **(B)** Holm oak (scale bar length: 60 μm).

**FIGURE 4:**
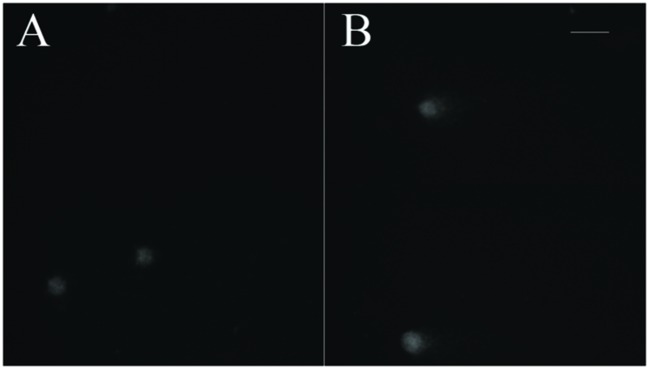
**Nucleoids of holm oak stained with ethidium bromide. (A)** Fourth leaf – control; **(B)** Fourth leaf – irradiated with 3 Gy X-rays (scale bar length: 100 μm).

The aim of the article is to present a method for the release of mesophyll protoplast from leaf tissues of *Q. ilex*. Currently, an efficient system for the isolation of oak protoplasts was obtained only from *in vitro* tissues, for instance, from shoots and callus from *Quercus acutissima* (Ide et al., 1991; Wakita et al., 1992). The leaf tissues from adult oak plants are considered recalcitrant material, even if collected soon after bud burst in *Quercus petraea* (Ahuja, 1984). Concerning the age of the leafs, the exposure to environmental constraints could lead to the increase of some compounds such as phenylpropanoids in plant tissues, as reported by Jones et al. (2012). In the same context, it is recently highlighted that abiotic stresses also can interact with cellulose synthesis altering cell wall composition (Wang et al., 2016). Even though several enzyme solutions were previously tested (data not shown), in the optimal results of the present work, the number and quality of isolated protoplasts in terms of fast digestion (4 h) were obtained using a solution starting from Wakita et al. (1992). In fact, in order to improve the digestion of the cell wall, trichomes were removed and lower epidermis were scratched, as well as 30 min of vacuum exposure was introduced when leaf tissue was dipped in the enzyme solution. Moreover, the success of the tested enzyme solution depends on the presence of cellulase Onozuka RS combined with macerozyme R-10 according to Conde and Santos in *Ulmus minor* (2006). **Figure 2** shows the digested holm oak leaf tissue (A) and the obtained protoplasts for holm oak (B) and white poplar (C), respectively. Immediately after isolation, the protoplasts (**Figure 3**) were counted using a hemocytometer and the results are presented in **Table 3**. *Q. ilex* fourth leaf yielded the highest amount of protoplasts while the *Q. ilex* 12th leaf yielded a 10th compared to the first one. This result demonstrates that the age of the leaf is a factor affecting protoplast isolation. In *Populus alba*, the quantity of isolated protoplasts was only slightly lower as compared to *Q. ilex* fourth leaf. Then, the obtained values are much higher than previously reported in the literature (Conde and Santos, 2006). Moreover, the isolated protoplasts were all viable in the species analyzed, as detected by the TBE assay. On the other hand, protoplasts isolated with purified cellulase exhibited low reactive oxygen species (ROS) levels that correlate with the highest viability in tobacco (Papadakis and Roubelakis-Angelakis, 2002). Although, the chromatin structure is subject to change during cell wall digestion (Feher, 2015), the fast isolation procedure set up in the present work leads to the undamaged nucleoids presented in **Figure 4A**. This confirms the absence of DNA damage in terms of single and double stranded breaks and alkali labile sites as well, while mechanical methods for nuclei isolations such as chopping may affect DNA integrity more. At present, the convenient number of nuclei to perform SCGE assay is under debate. Recently, it has been hypothesized that a number smaller than 50 records could cause a bias in outcomes for any statistical approach (Lanier et al., 2015). The whole process here is simple and efficient because more than 100 nuclei per slide were easily obtained. In order to test the efficiency of the isolation protocol for the SCGE assay, *Q. ilex* fourth leaf protoplasts were exposed to 3 Gy of X-ray and slides were prepared immediately after the irradiation. The results of the comet assay reported in **Table 4** clearly show a significant increase of tail moment, as well as tail intensity in the irradiated samples (**Figure 4B**).

**Table 3:** Mesophyll protoplast counting (average value ± standard error) after enzymatic digestion.

Source material	# prot. (10^6^)/2.5 ml ES	FL weight (mg)	# prot. (10^6^)/g leaf
*Quercus ilex –* fourth leaf (*N* = 6)	3.4 ± 0.3	60 ± 6.0	61.5 ± 9.7
*Q. ilex –* 12th leaf (*N* = 5)	0.4 ± 0.1	57 ± 5.0	7.4 ± 0.8
*Populus alba –* fourth leaf (*N* = 7)	1.2 ± 0.1	28 ± 2.6	45.7 ± 4.0

*prot., protoplasts; ES, enzyme solution; FL, fresh leaf*.

**Table 4:** Single cell gel electrophoresis (SCGE) assay in mesophyll protoplasts of *Q. ilex* (control and X-ray irradiated) *N* = 100.

Source material	Tail moment (arbitrary)	Tail intensity (%)	Tail length (μm)	Head intensity (%)	Head length (μm)
Fourth leaf – control	0.54 ± 0.08	4.46 ± 0.63	12.70 ± 0.61	95.54 ± 0.63	19.02 ± 0.54
Fourth leaf – 3 Gy	2.90 ± 0.15^∗^	33.04 ± 1.89^∗^	24.71 ± 1.40^∗^	66.96 ± 1.89	11.04 ± 0.60

*^∗^Student’s t-test, X-rays versus control, p < 0.01*.

In conclusion, 100s of reports on protoplast technology have been published, but the establishment of an efficient system for the isolation of mesophyll protoplasts from sclerophyll plants has still been lacking. The whole process here is simple and efficient to obtain protoplasts from young fresh leaf tissues of holm oak. In addition, the isolated protoplasts could be handled for comet assay technique, making it possible to detect the DNA damage in stressed plants of this recalcitrant species. Protoplasts technology coupled with SCGE assay could be suitable for the estimation of DNA integrity in holm oak, a sclerophyll plant with a wide distribution in natural Mediterranean ecosystems, as well as in urban areas subjected to environmental stress.

## Author Contributions

EK is the scientist responsible for the funding project, expert in forest biotechnology and a substantial contributor to the conception and design of the work, and to the acquisition and interpretation of the data for the work. RM is an expert in SCGE and a substantial contributor to the conception and design of the work, and to the acquisition and interpretation of the data for the work. ST is an expert in forest genetics and a substantial contributor to the conception and design of the work, acquisition, analyses, and interpretation of the data for the work. LP is involved in the acquisition, analyses and interpretation of the data for the work. She is the author of the artwork of the article, since she is a professional designer (Autocad^®^ 2016 from Autodesk^®^). CS is an expert in *in vitro* culture and protoplast isolation, a substantial contributor to the conception and design of the work, and he critically revised the work. ZC is involved in the acquisition, analyses and interpretation of the data. She provided for the drafting of the references section of the work. GS-M is involved in the scientific team of the funding project, a substantial contributor to the conception and design of the work. He also critically revised the work. All the authors approved the final version of the work to be published and are accountable for all aspects of the work in ensuring that questions related to the accuracy or integrity of any part of the work are appropriately investigated and resolved.

## Conflict of Interest Statement

The authors declare that the research was conducted in the absence of any commercial or financial relationships that could be construed as a potential conflict of interest.
